# Stromal vascular fraction inhibits renal fibrosis by regulating metabolism and inflammation in obstructive nephropathy

**DOI:** 10.3389/fphar.2025.1559446

**Published:** 2025-05-13

**Authors:** Guang Yue, Yunjie Yang, Hongshuai Jia, Yangyang Wu, Lifei Ma, Xiaoyu Yi, Yuandong Tao, Huixia Zhou

**Affiliations:** ^1^ Department of Pediatric Urology, The Seventh Medical Center of Chinese PLA General Hospital, Beijing, China; ^2^ Medical School of Chinese PLA, Beijing, China; ^3^ Department of Pediatric Surgery, The Sixth Affiliated Hospital, School of Medicine, South China University of Technology, Foshan, China

**Keywords:** SVF, obstructive nephropathy, renal fibrosis, inflammation, PPAR

## Abstract

Obstructive nephropathy is one of the leading causes of kidney injury and fibrosis, which can lead to end-stage renal disease (ESRD). Stromal vascular fraction (SVF), a heterogeneous cell mixture derived from adipose tissue, has been widely used for regenerative medicine across many preclinical models and clinical applications. Recent studies have suggested that SVF can alleviate acute kidney injury in mice. However, to our knowledge, the therapeutic effects of SVF on obstructive nephropathy have not been studied before. In this study, we evaluated the therapeutic potential of SVF on obstructive nephropathy in mice with unilateral ureteral obstruction (UUO). We revealed that autologous SVF administration mitigated UUO-induced renal fibrosis. SVF treatment inhibited both the infiltration of neutrophils and CD4^+^ T cells, as well as the production of inflammatory cytokines. Moreover, SVF promoted metabolic reprogramming and improved mitochondrial function in the obstructed kidneys, partially through PPAR pathway activation. Mechanistically, SVF-mediated PPAR activation inhibited the epithelial-mesenchymal transition (EMT) process of tubular cells, thus alleviating renal fibrosis in UUO mice. We further confirmed that pharmacological activation of PPAR pathway significantly reduced fibrosis in UUO kidneys. Therefore, our study suggests that SVF may represent a promising therapeutic strategy for obstructive nephropathy.

## Graphical Abstract

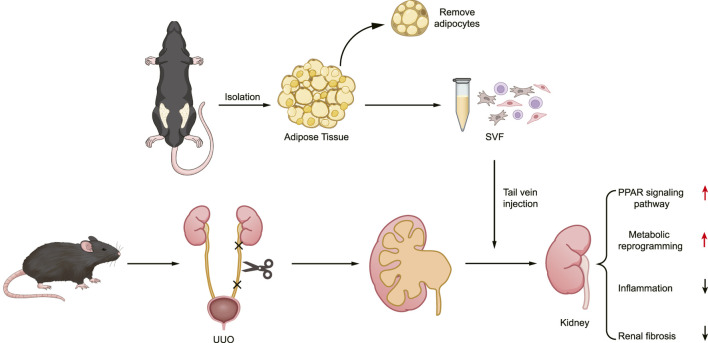

## 1 Introduction

Obstructive nephropathy, a common medical problem that affects many people, is a chronic and inflammatory process caused by urological obstruction. In infants and children, ureteropelvic junction obstruction (UPJO) is the most common cause of obstructive nephropathy (Stevens, 2018; Vemulakonda, 2021; Chevalier, 2015). In adults, most renal obstructions result from kidney stones, affecting approximately 10% of adults worldwide (Stevens, 2018). A review identified that 3.1% of individuals had hydronephrosis in 59,064 autopsies ranging from neonates to the elderly (Stevens, 2018). The obstruction decreases blood supply and triggers tubular epithelial cell damage and chronic inflammation, which subsequently leads to myofibroblast activation, excessive production of extracellular matrix, and renal fibrosis (Humphreys, 2018; Jackson et al., 2018). This may ultimately result in kidney function loss and end-stage renal disease (ESRD). Currently, aside from surgery to remove kidney stones or correct UPJO to prevent further injury, specific and effective therapies for obstruction-induced renal fibrosis are lacking.

Stromal vascular fraction (SVF) is a heterogeneous mixture of cells derived from adipose tissue (Guo et al., 2016). It contains stem/progenitor cells such as adipose-derived stem cells (ADSCs), stromal cells such as endothelial cells, fibroblasts, and pericytes, as well as immune cells such as macrophages and T cells (Goncharov et al., 2023; Al-Kharboosh et al., 2022). SVF has been widely used for regenerative medicine due to its potential to facilitate tissue repair (Koh et al., 2011; Bensemmane et al., 2023; Zhou et al., 2016), angiogenesis (Morris et al., 2015), and immunomodulation (Riordan et al., 2009; Dong et al., 2013). The therapeutic effect of SVF has been investigated in multiple preclinical models, including burn wounds, diabetic foot ulcers, acute myocardial infarction, and numerous others (Atalay et al., 2014; Cianfarani et al., 2013; Premaratne et al., 2011). Moreover, increasing numbers of clinical studies have demonstrated improved outcomes with SVF therapy in patients with ischemic stroke, myocardial ischemia, idiopathic pulmonary fibrosis, chronic liver failure, and knee osteoarthritis (Bateman et al., 2018; Andia et al., 2019; Vargel et al., 2022; Zhang et al., 2022). Notably, recent studies have shown that SVF administration could attenuate acute renal injury induced by ischemia-reperfusion and cisplatin (Zhou et al., 2016; Yasuda et al., 2012). However, to our knowledge, few studies have addressed the preventive effects of SVF against the development of chronic kidney disease, such as obstructive nephropathy.

In this study, we sought to investigate the therapeutic role of SVF in obstructive nephropathy using the unilateral ureteral obstruction (UUO) model. Our findings demonstrate that SVF significantly attenuated obstruction-induced renal fibrosis and inflammatory responses. Mechanistically, the therapeutic efficacy correlated with metabolic reprogramming and the activation of the PPAR signaling pathway in UUO kidneys.

## 2 Materials and methods

### 2.1 SVF isolation and detection

SVF was extracted from C57BL/6 mouse inguinal subcutaneous adipose tissue as previously described (Liu et al., 2023). C57BL/6 mice were procured from Spfbiotech (Beijing) and housed in specific-pathogen-free (SPF) conditions. The tissue was washed with ice-cold sterile PBS, diced, and subjected to enzymatic digestion using 0.075% type I collagenase at 37°C for 45 min. The tissue was then filtered through a 100 μm nylon mesh and centrifuged at 500 g for 5 min. The resultant cells were suspended at a density of 2 × 10^7^/mL in PBS and administered via the tail vein (100μL/mouse). Flow cytometry was used to detect the composition of SVF using a FACSCanto II (BD Biosciences). The following fluorescent antibodies were used (all from BioLegend): CD31-PE (102407), CD90-APC (140311), CD45-BV421 (103134), CD11b-FITC (101206), CD11c-PE/Cyanine7 (117317), and CD29-APC/Cyanine7 (102225).

### 2.2 Mice and study design

Ethical approval for the animal experimental protocols was obtained from the Ethical Committee of Chinese PLA General Hospital. Eight-week-old male mice were used for the UUO model. After anesthesia (Avertin, Sigma, T48402), a midline abdominal incision was made and the left ureter was double ligated. The UUO mice were randomly assigned to two groups (n = 5 per group). The experimental group received intravenous SVF injections on days 3 and 6 post-UUO surgery. On day 14, mice were euthanized and kidneys were collected for analysis.

### 2.3 Masson staining and histological analysis

The murine kidneys were fixed in 4% paraformaldehyde and embedded in paraffin. Renal sections were stained with Masson’s trichrome. Images were obtained using a NanoZoomer Slide Scanner (Hamamatsu Photonics).

### 2.4 Renal function analysis

Mouse serum was collected via centrifugation, and blood urea nitrogen (BUN) and creatinine levels were quantified using a Urea Assay Kit (C013-2-1) and a Creatinine Assay Kit (C011-2-1, Nanjing Jiancheng, China) following the manufacturer’s protocol.

### 2.5 Western blots

Renal protein extracts were prepared following standard protocols. The tissue lysates were separated by SDS-PAGE and transferred to polyvinylidene difluoride membranes (Millipore). The following primary antibodies were used: anti-β-tubulin (Huaxingbio, HX1829), anti-GAPDH (Proteintech, 60004-1-Ig), anti-fibronectin (Proteintech, 66042-1-Ig), anti-Col I (Abcam, ab260043), anti-αSMA (Proteintech, 67735-1-Ig), anti-NF-κB p65 (CST, 8242T), anti-PPARα (Huaxingbio, HX18360), anti-PPARγ (Proteintech, 16643-1-AP), anti-E-Cadherin (CST, 3195T), and anti-N-Cadherin (CST, 13116S). Western blot quantification was performed using ImageJ software.

### 2.6 Real-time quantitative PCR (qPCR)

Murine kidneys were homogenized and total RNA was extracted using an RNA Extraction Kit (Huaxingbio, HXR8075) following the manufacturer’s protocol. Complementary DNA (cDNA) was synthesized using the Reverse Transcription Kit (Takara, RR037A). Real-time quantitative PCR was performed on an iQ5 Real-Time PCR Detection System (Bio-Rad). The expression of the target gene was normalized to the housekeeping gene *Gapdh*. Relative gene expression was calculated via the standard 2^−ΔΔCT^ method. The qPCR primers are provided in Table 1.

**TABLE 1:** *qPCR* primers.

Genes	Forward	Reverse
*Gapdh*	AGG​TCG​GTG​TGA​ACG​GAT​TTG	TGT​AGA​CCA​TGT​AGT​TGA​GGT​CA
*Fn1*	CCC​TAT​CTC​TGA​TAC​CGT​TGT​CC	TGC​CGC​AAC​TAC​TGT​GAT​TCG​G
*Col1a2*	GCA​GGT​TCA​CCT​ACT​CTG​TCC​T	CTT​GCC​CCA​TTC​ATT​TGT​CT
*Acta2*	ACT​GCC​GAG​CGT​GAG​ATT​GT	TGA​TGC​TGT​TAT​AGG​TGG​TTT​CG
*Il1b*	TGT​AAT​GAA​AGA​CGG​CAC​ACC	TCT​TCT​TTG​GGT​ATT​GCT​TGG
*Il6*	TAC​CAC​TTC​ACA​AGT​CGG​AGG​C	CTG​CAA​GTG​CAT​CAT​CGT​TGT​TC
*Tnf*	TCCAGGCGGTGCCTATGT	CAC​CCC​GAA​GTT​CAG​TAG​ACA​GA
*Mrc1*	GTT​CAC​CTG​GAG​TGA​TGG​TTC​TC	AGG​ACA​TGC​CAG​GGT​CAC​CTT​T
*Retnla*	CAAGGAACTTCTTGCCAATCCAG	CCAAGATCCACAGGCAAAGCCA
*Cdh1*	CAGTTCCGAGGTCTACACCTT	TGAATCGGGAGTCTTCCGAAAA
*Tgfb1*	CGC​AAC​AAC​GCC​ATC​TAT​GA	ACT​GCT​TCC​CGA​ATG​TCT​GA

### 2.7 Bulk RNA sequencing (RNA-seq)

UUO kidneys from mice treated with or without SVF were subjected to RNA-seq analysis. RNA was extracted from the kidneys using TRIzol reagent (Thermo Fisher). RNA quality and quantity was qualified and quantified using a NanoDrop spectrophotometer and an Agilent 2100 Bioanalyzer. Subsequently, RNA was amplified and reverse-transcribed into cDNA for library construction. Sequencing was performed on an Illumina NovaSeq X Plus platform (Novogene, China). Raw sequencing data were aligned to the murine reference genome (version mm10).

### 2.8 Kidney leukocyte isolation and flow cytometry analysis

Renal tissues were enzymatically digested with 0.05% collagenase IV supplemented with 2 mM CaCl_2_ at 37°C for 25 minutes as previously described (Tao et al., 2023). The digested tissue was filtered through a 100 μm nylon mesh. The cell suspension was centrifuged at 500 g for 5 minutes and then incubated with an Fcγ receptor blocker (101320, BioLegend) for 10 minutes. The following fluorescent antibodies (all from BioLegend) were used: CD45-BV421 (103134), CD11b-FITC (101206), Ly6G-APC/Cyanine7 (127624), Ly6C-PE (128008), F4/80-APC (123116), CD206-PE/Cyanine7 (141720), CD3-PE (100206), CD4-PE/Cyanine7 (116016), CD8a-APC/Cyanine7 (100713), NK1.1-FITC (156508), and CD20-APC (152107). Flow cytometry was performed using a FACSCanto II (BD Biosciences). The data were analyzed using FlowJo software 10.4.

### 2.9 Cell culture and treatment

The murine tubular cell line TCMK-1 was acquired from the Cell Resource Center, Institute of Basic Medical Sciences (Beijing, China). The cells were cultured in RPMI 1640 medium (Gibco) supplemented with 10% fetal bovine serum (FBS). Tubular EMT was induced with recombinant human TGF-β1 (MCE, HY-P7118, 10 ng/mL). The PPARγ agonist pioglitazone (S2590, Selleck) and antagonist T0070907 (S2871, Selleck) were used.

### 2.10 Statistical analysis

Statistical analyses were performed using GraphPad Prism (version 10.1.2). Data are presented as mean ± standard error of the mean (SEM). Experiments were replicated at least three times. The two-tailed Student’s t-test was used for two-group comparisons. One-way analysis of variance (ANOVA) was used to assess comparisons among three or more groups. A p-value less than 0.05 was considered statistically significant.

## 3 Results

### 3.1 Characterization of murine SVF

**FIGURE 1:**
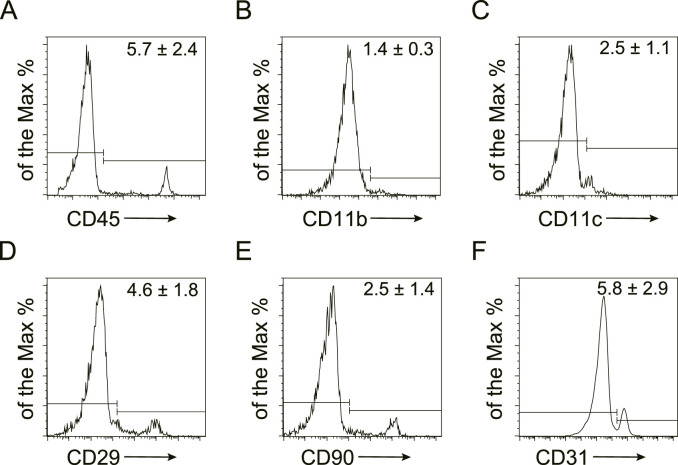
Characterization of murine SVF by flow cytometry Representative flow cytometry plots of murine stromal vascular fraction (SVF). The expression of hematopoietic (CD45, CD11b, and CD11c), mesenchymal (CD29, CD90), and endothelial (CD31) markers were detected and the quantification was shown as the mean ± SEM.

To characterize the cellular composition of SVF, flow cytometry was performed on freshly isolated stromal vascular fraction (Figure 1). Quantification identified the following marker expression profiles: hematopoietic lineage: CD45 (5.7% ± 2.4%), CD11b (1.4% ± 0.3%), and CD11c (2.5% ± 1.1%); mesenchymal markers: CD29 (4.6% ± 1.8%) and CD90 (2.5% ± 1.4%); and endothelial marker: CD31 (5.8% ± 2.9%). These data confirm the heterogeneous cellular composition of SVF, encompassing hematopoietic, stromal, and vascular components.

### 3.2 SVF alleviated UUO-induced renal fibrosis and injury

**FIGURE 2:**
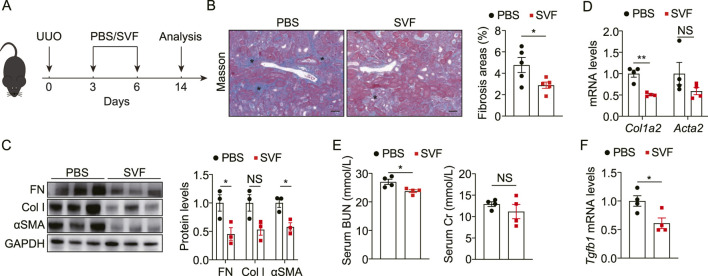
SVF alleviated UUO-induced renal fibrosis and injury **(A)** Experimental design was shown. **(B)** Representative Masson staining and quantification of UUO kidneys from control and SVF-treated mice (n = 5), scale bar = 40 μm. Asterisks (*) indicate regions of interstitial collagen deposition (in blue). **(C)** Immunoblots and quantification of FN, Col I, and αSMA expression in UUO kidneys from control and SVF-treated mice (n = 3). **(D)** qPCR analysis for *Col1a2* and *Acta2* in UUO kidneys from control and SVF-treated mice (n = 4). **(E)** Serum levels of BUN and Cr in control and SVF-treated mice (n = 4). **(F)** qPCR analysis for *Tgfb1* in UUO kidneys from control and SVF-treated mice (n = 4). The results represent mean ± SEM. *p < 0.05, **p < 0.01, NS no significant.

To investigate the potential of SVF to alleviate obstructive nephropathy, we employed the UUO mouse model (Figure 2A). Compared with controls, SVF-treated mice exhibited reduced collagen deposition in UUO kidneys as evidenced by Masson staining (Figure 2B). Notably, SVF administration resulted in significant downregulation of fibrotic markers, including fibronectin (FN), collagen I (Col I), and α-smooth muscle actin (αSMA), at both the protein and transcript levels (Figures 2C,D), indicating suppression of obstruction-induced renal fibrosis. Consistent with histological improvements, serum blood urea nitrogen (BUN) levels were also lower in SVF-treated mice versus controls (Figure 2E), suggesting renal functional preservation. However, serum creatinine levels remained comparable between two groups (Figure 2E). Importantly, SVF treatment significantly attenuated expression of transforming growth factor-β (TGF-β), a master regulator of fibrogenesis (Figure 2F). Collectively, these findings demonstrate SVF exerts renoprotective effects in UUO-induced nephropathy.

### 3.3 SVF contributed to metabolic reprogramming and reduced inflammation in UUO kidneys

**FIGURE 3:**
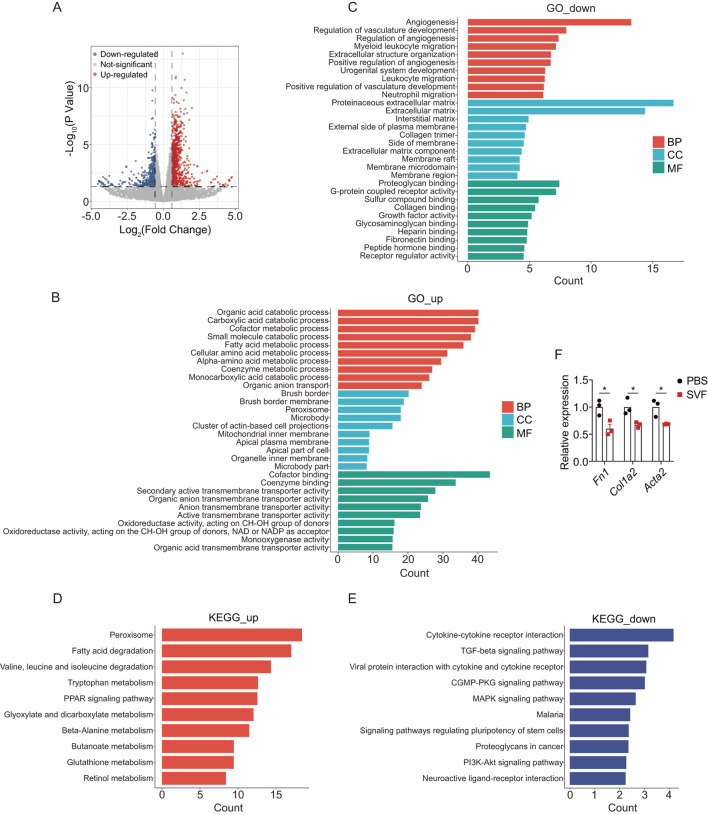
SVF contributed to metabolic reprogramming and reduced inflammation in UUO kidneys **(A)** Volcano plot showing the differential expressed genes in UUO kidneys between control and SVF-treated mice (fold change >1.5, p < 0.05). GO enrichment analysis of **(B)** the upregulated and **(C)** the downregulated genes in SVF-treated mice. KEGG enrichment analysis of **(D)** the upregulated and **(E)** the downregulated genes in SVF-treated mice. **(F)** The expression levels of *Fn1*, *Col1a2*, and *Acta2* in control and SVF-treated mice as revealed by RNA-seq (n = 3). The results represent mean ± SEM. *p < 0.05.

To elucidate the mechanisms underlying SVF-mediated renal protection, bulk RNA sequencing (RNA-seq) was performed on UUO kidneys from SVF-treated versus control mice. Differential expression analysis identified 716 upregulated and 437 downregulated genes in SVF group (fold change >1.5, p < 0.05) (Figure 3A). Gene ontology (GO) analysis revealed that upregulated genes in SVF-treated mice were significantly enriched in metabolic pathways, specifically organic acid metabolism, carboxylic acid metabolism, small molecule metabolism, fatty acid metabolism, and cellular amino acid metabolism (Figure 3B). These genes were further associated with brush border and mitochondrial inner membrane components (Figure 3B), suggesting that SVF may promote mitochondrial function in tubular cells. Conversely, downregulated genes were enriched in angiogenesis, immune cell migration, and extracellular structure organization (Figure 3C), indicating SVF-mediated suppression of inflammatory responses and fibrotic matrix deposition.

Subsequently, Kyoto Encyclopedia of Genes and Genomes (KEGG) pathway analysis was performed to identify signaling pathways modulated by SVF treatment. Consistent with GO findings, metabolic pathways, including peroxisome, fatty acid degradation, tryptophan metabolism, and PPAR signaling, ranked among the top enriched pathways for upregulated genes (Figure 3D). Conversely, downregulated genes were enriched in cytokine-cytokine receptor interactions, TGF-β signaling, MAPK signaling, and PI3K-Akt signaling (Figure 3E). Notably, TGF-β is a well-characterized master regulator of organ fibrosis (Meng et al., 2016), while PI3K-Akt pathway promotes kidney fibrosis by regulating collagen deposition and epithelial-mesenchymal transition (EMT) (Hu et al., 2021). Moreover, RNA-seq analysis revealed that the SVF-treated group exhibited significantly reduced expression levels of *Fn1*, *Col1a2*, and *Acta2*, suggesting that SVF may attenuate renal fibrosis progression (Figure 3F). These data imply that SVF attenuates renal fibrosis, at least partially, by suppressing pro-fibrotic pathways (e.g., TGF-β/PI3K-Akt) in UUO kidneys.

### 3.4 SVF inhibited renal inflammation in UUO kidneys

**FIGURE 4:**
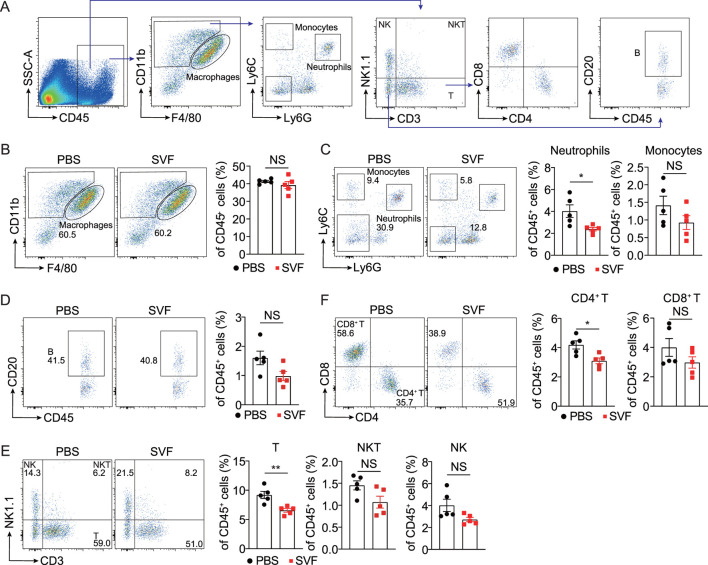
SVF inhibited the accumulation of neutrophils and CD4^+^ T cells in UUO kidneys **(A)** Gating strategy of kidney immune cells. Representative flow cytometry plots and quantification of **(B)** macrophages, **(C)** neutrophils and monocytes, **(D)** B cells, **(E)** T, NK, and NKT cells, and **(F)** CD4^+^ T and CD8^+^ T cells in UUO kidneys from control and SVF-treated mice (n = 5). The results represent mean ± SEM. *p < 0.05, **p < 0.01, NS no significant.

Inflammation plays a central role in the pathogenesis of kidney injury and fibrosis across etiologies (Tang et al., 2019). To characterize immune infiltration in UUO kidneys, we performed flow cytometric analysis of immune cell populations, including macrophages, neutrophils, monocytes, T cells, natural killer T (NKT) cells, natural killer (NK) cells, and B cells (Figure 4A). Notably, SVF treatment significantly reduced renal infiltration of neutrophils and CD4^+^ T cells (Figures 4B–F). This finding aligns with established evidence implicating neutrophils and CD4^+^ T cells as key mediators of fibrogenesis in UUO models (Ryu et al., 2022; Tapmeier et al., 2010). Collectively, these data demonstrate that SVF suppresses the accumulation of pro-fibrotic immune subsets in obstructed kidneys.

**FIGURE 5:**
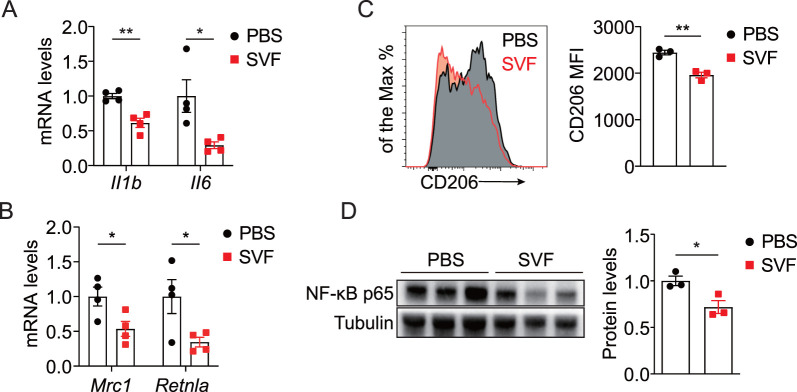
SVF inhibited UUO-induced kidney inflammation **(A,B)** qPCR analysis for *Il1b*, *Il6*, *Mrc1*, and *Retnla* expression in UUO kidneys from control and SVF-treated mice (n = 4). **(C)** Representative plots and quantification of CD206 expression in renal macrophages from control and SVF-treated mice (n = 3). **(D)** Immunoblots and quantification of NF-κB p65 expression in UUO kidneys from control and SVF-treated mice (n = 3). The results represent mean ± SEM. *p < 0.05, **p < 0.01.

Next, we evaluated kidney inflammation by analyzing inflammatory cytokines and pathways in UUO kidneys. Consistent with RNA-seq data, SVF treatment significantly inhibited pro-fibrotic cytokines IL-1β and IL-6 (Figure 5A). M2 macrophages are recognized as critical mediators of renal fibrosis through pro-fibrotic factors and macrophage-myofibroblast transition (MMT) (Tang et al., 2019). We observed that SVF treatment reduced expression levels of M2 markers *Mrc1* and *Retnla* in UUO kidneys (Figure 5B). Additionally, CD206 expression in renal macrophages exhibited a marked decrease in SVF-treated mice (Figure 5C). Furthermore, NF-κB p65 levels were significantly lower in SVF-treated mice compared to controls (Figure 5D). These results indicate that SVF administration inhibits UUO-induced kidney inflammation.

### 3.5 SVF contributed to PPAR activation and inhibited EMT in tubular cells

**FIGURE 6:**
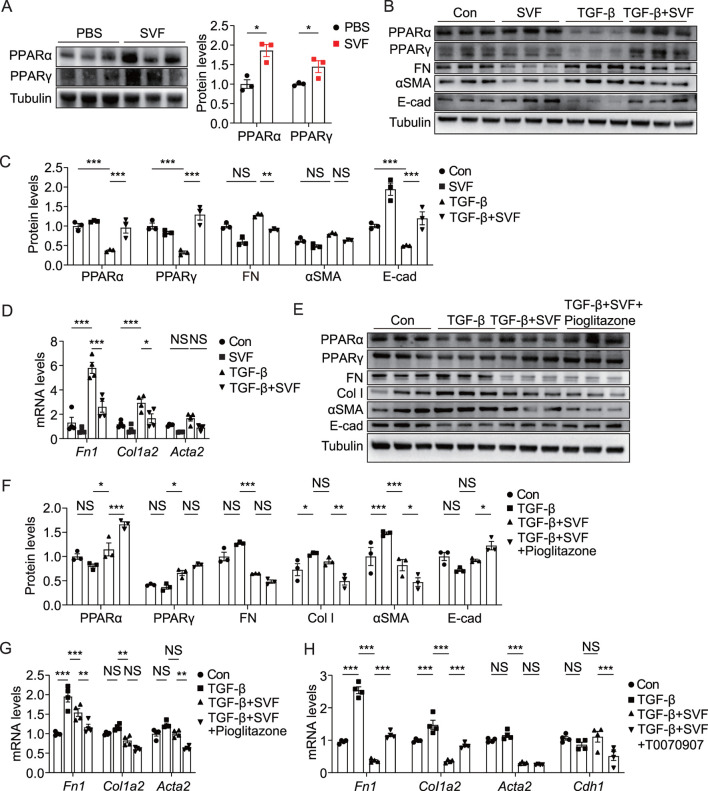
SVF promoted PPAR activation and inhibited EMT in TCMK-1 cells **(A)** Immunoblots and quantification of PPARα and PPARγ in UUO kidneys from control and SVF-treated mice (n = 3). **(B)** Immunoblots and **(C)** quantification of PPARα, PPARγ, FN, αSMA, and E-cad in TCMK-1 cells treated with or without SVF (n = 3). **(D)** qPCR analysis for *Fn1*, *Col1a2*, and *Acta2* in TCMK-1 cells treated with or without SVF (n = 4). **(E)** Immunoblots and **(F)** quantification of PPARα, PPARγ, FN, Col I, αSMA, and E-cad in TCMK-1 cells treated with SVF or SVF and pioglitazone combined (n = 3). **(G)** qPCR analysis for *Fn1*, *Col1a2*, and *Acta2* in TCMK-1 cells treated with SVF or SVF and pioglitazone combined (n = 4). **(H)** qPCR analysis for *Fn1*, *Col1a2*, *Acta2*, and *Cdh1* in TCMK-1 cells treated with SVF or SVF and T0070907 combined (n = 4). The results represent mean ± SEM. *p < 0.05, **p < 0.01, ***p < 0.001, NS no significant.

Given that SVF upregulated the PPAR signaling pathway (Figure 3D) and PPAR plays a critical role in cellular metabolism (Gao and Gu, 2022), we sought to assess its impact on the PPAR pathway in both UUO kidneys and tubular cells. Our data revealed elevated levels of PPARα and PPARγ in obstructed kidneys from SVF-treated mice (Figure 6A). To further investigate this phenomenon, we examined SVF’s effects on TCMK-1 cells, a murine tubular cell line. Consistent with previous reports, recombinant human TGF-β significantly reduced PPARα and PPARγ expression (Figures 6B,C). Remarkably, SVF treatment restored PPARα and PPARγ levels in TCMK-1 cells (Figures 6B,C), indicating its PPAR signaling activation potential. Importantly, SVF counteracted the TGF-β-mediated EMT process as evidenced by reduced FN and αSMA expression alongside increased epithelial marker E-cadherin (Figures 6B–D). These findings collectively suggest that SVF attenuates EMT through PPAR signaling in tubular cells.

To investigate whether PPAR signaling mediated SVF’s inhibitory effect on tubular EMT, we employed a well-characterized PPARγ agonist pioglitazone and specific PPARγ antagonist T0070907 (Qian et al., 2022; Nian et al., 2024). When combined with SVF treatment, pioglitazone produced greater suppression of Col I and αSMA while enhancing E-cadherin levels (Figures 6E–G), confirming a critical role of PPAR activation in EMT inhibition. Conversely, pharmacological PPARγ blockade with T0070907 significantly restored the expression of FN and Col I while diminishing the levels of E-cadherin (*Cdh1*) in SVF-treated TCMK-1 cells, demonstrating that PPARγ inactivation counteracts SVF-mediated EMT suppression (Figure 6H). These findings establish that SVF attenuates tubular EMT through at least partial activation of the PPAR signaling pathway.

### 3.6 PPAR activation alleviated UUO-induced renal fibrosis and inflammation

**FIGURE 7:**
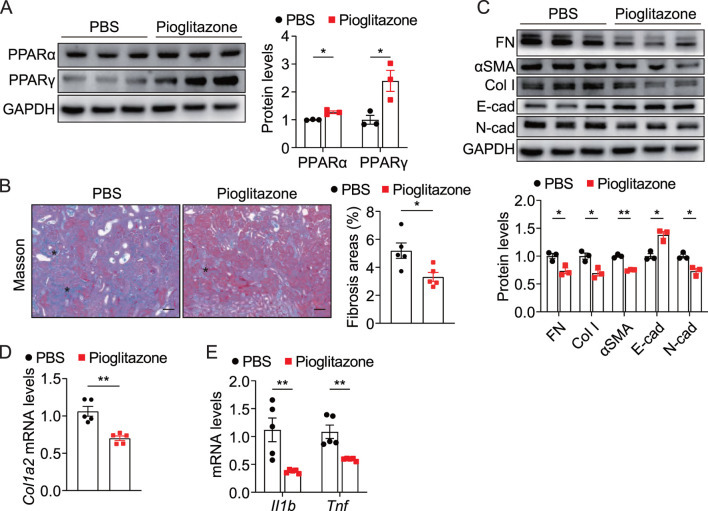
Pharmacological activation of PPAR alleviated UUO-induced renal fibrosis and inflammation **(A)** Immunoblots and quantification of PPARα and PPARγ in UUO kidneys from control and pioglitazone-treated mice (n = 3). **(B)** Representative Masson staining and quantification of UUO kidneys from control and pioglitazone-treated mice (n = 5), scale bar = 40 μm. Asterisks (*) indicate regions of interstitial collagen deposition (in blue) **(C)** Immunoblots and quantification of FN, Col I, αSMA, E-cad, and N-cad expression in UUO kidneys from control and pioglitazone-treated mice (n = 3). **(D,E)** qPCR analysis for *Col1a2*, *Il1b*, and *Tnf* in UUO kidneys from control and pioglitazone-treated mice (n = 5). The results represent mean ± SEM. *p < 0.05, **p < 0.01.

To investigate the role of PPAR activation in obstruction-induced renal fibrosis, we administered the PPARγ agonist pioglitazone to UUO mice. As expected, pioglitazone administration significantly increased the expression of PPARα and PPARγ in UUO kidneys (Figure 7A). Histopathological assessment revealed that pioglitazone administration attenuated collagen deposition in pioglitazone-treated mice compared to controls (Figure 7B). Furthermore, pioglitazone-treated mice displayed reduced levels of the fibrosis markers FN, αSMA, Col I, and N-cadherin while increasing levels of the epithelial marker E-cadherin (Figures 7C,D), which was consistent with *in vitro* studies in tubular cells. Notably, pioglitazone also markedly suppressed inflammatory cytokine production in obstructed kidneys, including IL-1β and TNF (Figure 7E). These data demonstrate that pharmacological PPAR activation via pioglitazone effectively mitigates both renal fibrosis and inflammation in UUO mice.

## 4 Discussion

Our study demonstrated that autologous SVF administration attenuates obstructive nephropathy in mice. SVF intervention improves metabolic processes, inhibits inflammatory cytokine production, and reduces neutrophils and CD4^+^ T cell infiltration in UUO kidneys. Importantly, we further identified that SVF significantly inhibits tubular EMT and renal fibrosis through, at least in part, activation of the PPAR signaling axis.

SVF constitutes a heterogeneous cell population comprising ADSCs and other regenerative components. Similar to mesenchymal stem cells (MSCs), ADSCs have been widely studied and exploited for the treatment of tissue injury due to their regenerative, proangiogenic, anti-fibrotic, and immunomodulatory properties (Guo et al., 2016). ADSCs can differentiate into tissue functioning cells because of their pluripotent capabilities, such as neonatal adipocytes in fat grafts (Zhu et al., 2015). Beyond direct differentiation, SVF can also induce host cell proliferation to regenerate injured tissue, such as fibroblasts in diabetic foot ulcers and nerve cells in peripheral nerve lesions (Han et al., 2010; di Summa et al., 2010). Importantly, accumulating evidence indicates that ADSCs drive neovascularization via angiogenic factor secretion, a critical mechanism for tissue regeneration in pathologies including myocardial infarction, thermal injuries, diabetic ulcers, and ischemic myopathy (Atalay et al., 2014; Cianfarani et al., 2013; Li et al., 2013). This aligns with reports documenting that SVF facilitates angiogenesis by inducing VEGF production (Rehman et al., 2004). Interestingly, our transcriptomic analysis revealed unexpected downregulation of angiogenesis-related pathways in SVF-treated mice, a finding requiring further mechanistic validation through functional assays.

SVF is recognized to exert multifaceted immunomodulatory effects, mediated through three principal cellular components: ADSCs, macrophages, and regulatory T (Treg) cells (Gandolfi et al., 2023). Mounting evidence highlights the immunomodulatory effects of MSCs (including ADSCs), although the associated mechanisms are not fully understood (Ketterl et al., 2015; Bowles et al., 2017). Adipose-resident macrophages predominantly exhibit an anti-inflammatory M2 phenotype, characterized by the production of IL-10 and other immunosuppressive mediators (Lumeng et al., 2007; Zeyda et al., 2007). Notably, ADSCs have been shown to actively promote M2 macrophage polarization (Shang et al., 2015). Furthermore, SVF contains detectable populations of Treg cells, a specialized lymphocyte subset that suppress inflammation via secretion of immunoregulatory cytokines (Wang et al., 2024). Our experimental data revealed that SVF significantly reduced the accumulation of neutrophils and CD4^+^ T cells, as well as the production of inflammatory cytokines in UUO kidneys. However, the precise interplay between these cellular components in mediating SVF’s immunomodulatory effects needs systematic investigation.

Emerging evidence demonstrates the therapeutic potential of SVF in combating fibrotic pathologies. For example, autologous SVF exerts anti-fibrotic effects in ischemia-reperfusion induced fibrosis in kidney and heart, and also prevents the development of fibrosis in the tunica albuginea in a rat model of Peyronie’s disease (Zhou et al., 2016; Castiglione et al., 2019). Furthermore, clinical studies confirm that autologous SVF injection prevents fibrosis of the corpus cavernosum caused by cavernous nerve injury (Qiu et al., 2012). Here, our findings provide experimental evidence that autologous SVF administration represents a novel therapeutic approach to alleviate renal fibrosis in obstructive nephropathy.

PPAR isoforms are ubiquitously expressed across renal cell populations, including proximal tubular cells, collecting duct epithelia, podocytes, and mesangial cells, to maintain energy homeostasis (Gao and Gu, 2022). PPAR target genes orchestrate fatty acid metabolism and inflammatory responses. Accumulating evidence established that all PPAR members (PPARα, PPARβ/δ, and PPARγ) are implicated in the pathogenesis of kidney diseases (Gao and Gu, 2022). Deletion of each of the three PPARs results in more severe kidney injury in murine models (Iwaki et al., 2019; Toffoli et al., 2017; Mukundan et al., 2009). Therefore, the PPAR pathway has emerged as a promising target for the treatment of kidney diseases. We showed that SVF infusion led to elevated levels of both PPARα and PPARγ in UUO kidneys, suggesting that SVF may induce the activation of PPAR signaling. However, the molecular mechanisms by which SVF activates the PPAR pathway remain to be elucidated. Besides, our study did not evaluate SVF’s effects on PPARβ/δ.

Our study has several limitations. First, the precise mechanisms through which SVF modulates mitochondrial function and metabolic reprogramming in tubular cells remain to be systematically investigated. Specifically, whether SVF-derived cellular components or paracrine factors mediate these effects requires functional validation through mitochondrial stress assays and metabolomic profiling. Second, while we identified PPARα/γ activation as a key pathway, the precise mechanisms driving SVF-induced PPAR signaling activation remain incompletely characterized. Notably, the potential involvement of PPARβ/δ isoform-specific effects needs further investigation. Third, the critical unresolved question of whether cellular (e.g., ADSCs, Tregs) or acellular components (e.g., matrix proteins, microRNAs) mediate the observed anti-fibrotic effects demands rigorous characterization. Future studies employing single-cell RNA sequencing of SVF subpopulations coupled with functional fractionation studies will be essential to delineate therapeutically active components.

In summary, we demonstrated that autologous SVF administration may mitigate renal inflammation and promote kidney metabolism in obstructive nephropathy. SVF mediates the activation of the PPAR pathway and inhibits the tubular EMT process, thus alleviating renal fibrosis in obstructed kidneys. Our study suggests that SVF may represent a promising strategy for obstructive nephropathy.

## Data Availability

The datasets presented in this study can be found in online repositories. The names of the repository/repositories and accession number(s) can be found below: https://www.ncbi.nlm.nih.gov/geo/, GSE281130.
